# Immunological Dysfunction in Tourette Syndrome and Related Disorders

**DOI:** 10.3390/ijms22020853

**Published:** 2021-01-16

**Authors:** Chia-Jui Hsu, Lee-Chin Wong, Wang-Tso Lee

**Affiliations:** 1 Department of Pediatrics, National Taiwan University Hospital Hsin-Chu Branch, Hsinchu 300, Taiwan; jry730701@gmail.com; 2 Department of Pediatrics, Cathay General Hospital, Taipei 106, Taiwan; leechinx@hotmail.com; 3 Graduate Institute of Clinical Medicine, National Taiwan University College of Medicine, Taipei 100, Taiwan; 4 Department of Pediatric Neurology, National Taiwan University Children’s Hospital, Taipei 100, Taiwan; 5 Department of Pediatrics, National Taiwan University College of Medicine, Taipei 100, Taiwan; 6 Graduate Institute of Brain and Mind Sciences, National Taiwan University College of Medicine, Taipei 100, Taiwan

**Keywords:** Tourette syndrome, PANDAS, immunological dysfunction, basal ganglia, neuroinflammation

## Abstract

Chronic tic disorder and Tourette syndrome are common childhood-onset neurological diseases. However, the pathophysiology underlying these disorders is unclear, and most studies have focused on the disinhibition of the corticostriatal–thalamocortical circuit. An autoimmune dysfunction has been proposed in the pathogenetic mechanism of Tourette syndrome and related neuropsychiatric disorders such as obsessive–compulsive disorder, autism, and attention-deficit/hyperactivity disorder. This is based on evidence from animal model studies and clinical findings. Herein, we review and give an update on the clinical characteristics, clinical evidence, and genetic studies in vitro as well as animal studies regarding immune dysfunction in Tourette syndrome.

## 1. Introduction

Gilles de la Tourette syndrome (TS) is a childhood-onset developmental neurological disease characterized by motor and phonic tics that was first described by the French physician Georges Gilles de la Tourette in 1885 [1]. Tic disorders are brief, repetitive, involuntary movements or sounds, such as grimacing, face-making, shoulder-jerking, or throat-clearing. The tics are usually preceded by a premonitory urge and are transiently relieved after the tics. The presentation of motor tics or phonic tics varies wildly, which may range from rapid meaningless movements or sounds to purposeful behaviors or speeches. Sometimes, tics can be orchestral, which means that different tics can occur one-by-one in a specific order. Moreover, the severity of the tics may fluctuate in different hours, days, or months, creating a “waxing and waning” character [2]. The diagnostic criteria of TS are as follows: (1) the presence of at least two motor tics and one phonic tic; (2) symptoms starting before 18 years and persisting for more than 1 year; (3) symptoms are not secondary to other neurological diseases such as encephalitis, stroke, or other intracranial lesions [3]. In different studies, the prevalence rate of TS is 0.1–6%, with male predominance, and the estimated pooled prevalence rate of TS is 0.53% [4,5,6]. Moreover, more than two-thirds of patients with TS have comorbidities, including attention-deficit hyperactivity disorder (ADHD) or obsessive–compulsive disorder (OCD) [7]. Other comorbidities, such as emotional disorder, depression, migraine or sleep disorders, or other neuropsychiatric disorders, are not unusual in patients with TS [7,8]. Tic symptoms usually occur between the ages of 4 and 6 years and reach utmost severity between age 10 and 12 years. More than half of the patients will have reduced severity by adulthood [2]. Some pharmacologic or nonpharmacologic treatments help patients deal with the symptoms in their daily life [9]. However, tic-suppressing agents are all symptomatic treatments because the pathophysiology of TS is still not understood.

Even though the main affected brain region in TS is still controversial, most of the studies have pointed out the abnormality of the basal ganglia and the related corticostriatal–thalamocortical (CSTC) circuit [6,10,11,12,13]. Dopamine, as the main excitatory neurotransmitter of the CSTC circuit, is revealed to play a role in the pathophysiology of TS [14,15]. Increased dopamine D2 receptor binding in the caudate nucleus has also been mentioned in some studies, which have also suggested the dysregulation of the dopaminergic system in patients with TS [16,17]. However, the etiology of TS is very complex. Current studies have suggested a multifactorial etiology in TS, including genetic, environmental, and immunological factors that establish one’s neurobiological vulnerability to TS [18,19].

An increasing number of studies have emphasized immunological involvement in TS. The correlation of tic disorder and group A *Streptococcus* (GAS) infection has already been confirmed for decades [20]. Tics or other neuropsychiatric disorders such as OCD might occur or worsen after GAS infection. Patients with TS also had higher rates of being GAS-positive in the throat specimen culture and higher anti-streptolysin O titers [21]. In addition, one disease entity that is characterized by sudden onset of tics, associated with obsessive–compulsive manifestation, behavior, and personality change in children with streptococcal infection, has been recognized in past decades; it is termed “pediatric autoimmune neuropsychiatric disorders associated with streptococcal infections” (PANDAS) [22]. As different infectious pathogens other than streptococcus have been identified to also be associated with neuropsychiatric manifestations, these disease entities are now called “pediatric acute-onset neuropsychiatric syndrome” (PANS) [23].

On the other hand, patients with TS have been found to have increased inflammatory activities with increased serum levels of tumor necrosis factor-alpha (TNF-α) and interleukin (IL)-12 [24]. A significant increase in the positive oligoclonal band (OCB) detection rate was also reported in patients with TS [25], which suggests abnormal plasma cell function. The evidence on immunological involvement in TS has accumulated in recent years. Combining these results may help us understand the pathogenic mechanisms of TS. Therefore, we will briefly review the major findings regarding the immunological involvement of TS in different aspects.

## 2. Association between Infection and TS and Related Disorders

### 2.1. GAS Infection

#### 2.1.1. Sydenham’s Chorea (SC) and Pediatric Autoimmune Neuropsychiatric Disorders Associated with Streptococcal Infections (PANDAS)

The most relevant and extensively studied infectious culprit associated with TS and related disorders is GAS. GAS is a common pathogen of acute pharyngitis in children and adolescents, which accounts for 20–37% of all pediatric cases [26,27]. The aberrant immune process associated with the existence of host autoantibodies against GAS antigens, following infection, may lead to rheumatic fever, a postinfectious autoimmune disease with multiorgan involvement. SC, a neurological manifestation or a variant of rheumatic fever, is characterized by an abrupt onset of chorea that typically involves the face and extremities. Patients may also present with various behavioral issues such as anxiety, OCD, and emotional liability [28].

The association of GAS with TS-related disorders has gained attention since the 1990s. Swedo et al. [22] described 50 children with a phenotype that is relatively distinct from SC. These children presented with acute onset of tic disorder, OCD, and emotional liability following GAS infection. This phenotype was designated as PANDAS. The working criteria for the diagnosis of PANDAS include (1) the existence of OCD or tic symptoms, (2) prepubertal onset, (3) symptoms occurring intermittently or following a sawtooth course, (4) temporal relationship of OCD/tic symptoms to GAS infection, and (5) presence of other neurological findings such as hyperactivity or choreiform movements. However, whether these criteria can be used to designate a unique clinical entity remains controversial. First, patients with SC might also develop a certain degree of tics. In addition, as tics and chorea are both rapid, involuntary, sudden-onset movement disorders, they might be difficult to differentiate in some cases. Moreover, Criterion 4 requires a temporal relationship of OCD/tic symptoms to GAS infection, which is supported by findings from a large population-based study, reporting that children with streptococcal throat infection have an increased risk of developing OCD and TS [29]. However, prospective longitudinal studies have shown that clinical exacerbation did not necessarily match temporally to GAS infection [30,31].

#### 2.1.2. GAS and TS

Other than SC and PANDAS, evidence from clinical studies has suggested that GAS infection can act as a trigger factor or can have a disease-modifying role in TS [29,32,33,34]. In a Taiwanese nationwide population-based retrospective study, Wang et al. [35] showed an increased risk of TS and ADHD in 2596 children with GAS infection. This study corresponded to another population-based study performed in the United States, which showed that patients with OCD, TS, or tic disorder were more likely to have had a prior streptococcal infection before the onset of symptoms. Furthermore, those with recent multiple infections with GAS have an increased risk for TS [34]. In a prospective longitudinal study, patients with large fluctuation of symptoms in tics/OCD have persistently elevated streptococcal titers compared with those with a stable/remitting course [33], suggesting a disease-modifying role of GAS in TS. However, subsequent new GAS infections or immune markers do not predict clinical exacerbations [21,36].

### 2.2. Other Pathogens

A Danish large-scale nationwide population-based study found that both streptococcal throat infection and nonstreptococcal infection are associated with elevated risks of OCD and tic disorders [29]. This finding indicates that GAS is not the only pathogen that plays a role in the pathogenesis of TS. The reported pathogens include *Mycoplasma pneumoniae*, *Enterovirus* (EV), *Chlamydia pneumoniae*, *Borrelia burgdorferi*, *Toxoplasma gondii*, and even *human immunodeficiency virus* (HIV). However, evidence regarding the association among other infectious pathogens is limited and the results were based on a small number of cases. Mycoplasma infection has been considered to be associated with TS in case reports [37,38]. This finding was supported by a study that showed that patients with TS have a significantly higher IgA antibody titer against *Mycoplasma pneumoniae* than healthy controls. In addition, tic exacerbations in two children were associated with *Mycoplasma* infection, while the symptoms improved after treatment [39]. Other Taiwanese population-based studies have also shown that EV infection is significantly associated with a higher incidence of tic disorders. In addition, they found that children with allergic diseases, including allergic rhinitis or asthma, exhibited a significantly increased risk for subsequent tic disorders [40,41]. Antonelli et al. [42] described that episodes of worsening of tics occurred after discontinuations of antiretroviral drugs in a patient with HIV-1. That patient had an increase in HIV-1 viral load and a decrease in CD4^+^ cell count. Intriguingly, the symptoms significantly improved following the reintroduction of antiretroviral therapy. Furthermore, a 9-year-old boy with severe tic disorders had intrathecal production of *B. burgdorferi*-specific IgG antibodies, indicating neuroborreliosis. The tics resolved completely, along with a decrease in *Borrelia*-specific antibody titers, after treatment [43]. However, further large-scale studies focusing on underlying pathomechanisms are needed to validate the aforementioned findings.

## 3. Immune Dysregulation in TS

Both allergic and autoimmune diseases may arise from an impairment of the immune tolerance process. Clinical reports that linked allergic diseases to TS were based on population-based studies [44,45]. By using the National Health Insurance research database in Taiwan, Chang et al. [44] found that TS is associated with common allergic diseases, including rhinitis, asthma, dermatitis, and allergic conjunctivitis. This evidence supports the finding that immunological dysfunction may play a partial role in the pathogenesis of TS. However, the clinical response of TS to immunotherapy was inconsistent among studies. Although case reports or small case-series studies have shown improvement following immune-modulating therapies in TS, including intravenous immune globulin (IVIG) [46], cyclooxygenase-2 inhibitor [47], adrenocorticotropic hormone, and prednisone [48], a placebo-controlled study on adults with tics showed that IVIG failed to demonstrate significant improvement in adult tics [49].

### 3.1. Genetic Expression in TS

In current studies, there were only a few de-novo coding variants proven to be correlated with TS, including WWC1 (WW and C2 domain containing 1), CELSR3 (cadherin EGF LAG seven-pass G-type receptor 3), NIPBL (nipped-B-like), and FN1 (fibronectin 1) [50]. The WWC1 gene has a role in cell polarity, migration, and trafficking. The CELSR3 gene is also involved in cell polarity and is essential for axon pathfinding in the central nervous system of mice. The NIPBL gene has a critical function in cell meiosis and influences gene expression during development. The FN1 gene is involved in cell differentiation, migration, and cell adhesion [50,51]. Knowing the function of these genetic variants also provide some other avenues to discover the pathogenesis of TS. However, for immunological disturbance in TS, Ercan-Sencicek et al. reported a functional mutation of *Hdc* (histidine decarboxylase) in a two-generation family [52]. The *Hdc* gene has an important role in histamine synthesis. In *Hdc* knockout mice, increased tic-like behaviors such as excessive grooming were recorded [53]. Altered histamine synthesis might result in dysregulation of peripheral inflammation via microglia modulation [54]. This finding also highlights the role of microglia dysregulation in immunological dysfunction in TS patients.

Some genetic expressions have been proven to be correlated with the susceptibility of autoimmune dysfunction in TS. Some studies have mentioned the correlation between TS severity and gene expression in gamma aminobutyric acid (GABA), acetylcholine, and catecholamine pathways [55,56]. GABA-related gene expression in TS includes GABA-A receptor alpha 2, GABA-A receptor alpha 3, GABA-A receptor alpha 4, GABA-A receptor beta 1, GABA-A receptor rho 1, GABA-B receptor 2 (GABBR2), GABA receptor-associated protein (GABARAP), G protein-coupled receptor 156, and solute carrier family 6 member 1 [55]. The expression of GABARAP has shown a negative correlation with tic severity, whereas the expression of other genes has exhibited a positive correlation. GABA is the major inhibitory neurotransmitter in the brain, and altered GABAergic parvalbumin neurons have been observed in patients with TS [57]. Increased expression of genes related to GABA receptors may reflect endogenous compensation in TS. However, GABA and GABA receptors can also be detected in monocytes, macrophages, and lymphocytes. GABBR2 acts as a chemoattractant in neutrophils and the regulated inflammatory response during ischemia reperfusion [58].

In the cholinergic pathway, altered gene expressions also occur, including expressions of cholinergic receptor nicotinic alpha 1, cholinergic receptor nicotinic alpha 9, cholinergic receptor nicotinic alpha 10, cholinergic receptor nicotinic gamma, phospholipase D1 phosphatidylcholine-specific, solute carrier family 5 member 7, collagen-like tail subunit of asymmetric acetylcholinesterase, solute carrier family 33 member 1, and phosphate cytidylyltransferase 1 choline alpha. Acetylcholine receptors are expressed in GABAergic and dopaminergic neurons in the striatum; they regulate dopamine release [59]. Moreover, acetylcholine receptors are involved in the regulation of B- and T-lymphocytes [60]. Both mechanisms may explain the pathogenetic role of acetylcholine in TS.

Catecholamine-related gene expression has also been proven to be correlated with tic severity. Gunther et al. [56] found overexpression of dopamine receptor D2, histamine receptor H3, monoamine oxidase B, brain-derived neurotrophic factor, synaptosomal-associated protein 25 kDa, solute carrier family 6 member 4, and solute carrier family 22 member 3 in patients with TS. Dopamine was proved to be related to T-lymphocyte activation, chemotactic migration, and T-cell cytokine secretion [61]. Dysregulation of GABA, acetylcholine, and catecholamine pathways may have a direct influence on the dysfunction of the CSTC circuit, and they may interrupt the normal function of immune cells and result in TS indirectly. However, whether the altered expression of these genes is the cause or the consequence of TS is still unclear. Further evaluation is warranted to clarify these phenomena.

### 3.2. Aberrant Peripheral Immune Activities

#### 3.2.1. Alternation in Immune Cell Subset and Immunophenotyping

Immunological tolerance to self-antigens and allergens is maintained via multiple suppressive mechanisms. CD4(+)CD25(+) Treg is a key player in this complex process, and its suppressive function is crucial to control autoimmunity, allergic response, and inflammatory response to infectious pathogens and allergens. Aberrant function or decreased number of Treg cells is associated with allergic diseases and numerous autoimmune diseases, including type 1 diabetes, multiple sclerosis, systemic lupus erythematosus, myasthenia graves, or rheumatoid arthritis [62].

Kawikova et al. [63] demonstrated a significant decrease in the number of Treg cells in patients with moderate-to-severe TS symptoms. Further decrease in the number of Treg cells was also observed during symptom exacerbation. This could be partly related to the immunomodulatory effects of dopamine. TS has been hypothesized to have dopaminergic hyperactivity [64]. In addition to its essential role in neurotransmission, dopamine has regulatory effects on the immune response. Dopamine was observed to suppress Treg cells [65]. Dopamine receptors expressed on immune cells mediate the immunomodulating effects of dopamine [66]. Consistent with this explanation, Ferrari et al. [67] reported higher expression levels of DRD5 dopamine receptor mRNA in peripheral blood lymphocytes of patients with TS. Another possible explanation is that the proallergic inflammatory environment might skew Treg cells toward a reactive pathogenic phenotype [68], which is in line with clinical reports that children were associated with a higher risk of TS in allergic diseases [44].

Further support to increase peripheral immune activity was obtained from a pilot study of lymphocyte immunophenotyping. Moller et al. [69] reported significantly increased numbers of both CD69^+^ B-lymphocytes and CD95^+^ T-lymphocytes in adults with TS. CD69 is considered an early activation marker of lymphocytes [70], thereby suggesting increased B-cell activation. CD95 is important in activation-induced cell death in T-cells [71]. An increase in the number of CD95^+^ T-cells may reflect augmented eradication of activated cells from the peripheral T-cell pool, thereby indicating increased immune activity.

#### 3.2.2. Dysregulation of Effector Molecules and Immunoglobulin (Ig)

Release of both innate and adaptive immune cells is modulated by effector molecules, including cytokines, chemokines, and adhesive molecules. Leckman et al. [24] reported increased baseline plasma levels of proinflammatory cytokine TNF-α and IL-12 in children with TS and/or early-onset OCD. Interestingly, levels of these two cytokines further increased during periods of symptom exacerbation. In line with this finding, a later study also showed elevated levels of proinflammatory cytokines, including IL-17A, IL-6, IL-12, and TNF-α in pediatric patients with TS without OCD. Additionally, children who are medication-naïve showed higher TNFα levels than healthy controls [72]. However, studies have reported inconsistent findings [73,74], which showed a decrease or no significant difference in the level of inflammatory cytokines. Taken together, the proinflammatory immune response may be at least partly related to the pathogenesis of TS. However, inflammatory cytokine levels may be altered by age, sex, medications, and comorbidities. Further evidence on the presence of proinflammatory mechanisms in TS came from reports on the increase of plasma levels of neopterin [73,75]. Given that neopterin is a marker of cellular immune activation, whose production is induced by cytokine interferon-γ (INF-γ), the upregulation of neopterin in TS may reflect the activation of the T-cell immune response in these patients.

A few reports have also described dysregulation of Ig in patients with TS. Bos-Veneman et al. [76] presented a consistent decrease of IgG3 plasma levels and a trend for lower IgM levels in two independent samples (adults and children) of patients with TS. However, the Ig levels did not correlate with tic severity. IgG3 is pivotal in activating the classical complement cascade, promoting the destruction of microbial pathogens through the formation of membrane attack complexes. Decreased IgG3 levels may lead to a defective immune response to pathogens, resulting in persistent inflammation. Additionally, Kawikova et al. [77] observed reduced plasma IgA levels in adults with TS and/or OCD, particularly in the PANDAS subgroup, thus implying that mucosal immunity may be impaired in these patients. However, whether this finding could apply to TS, in general, remains unclear and warrants further investigation.

### 3.3. Aberrant Neuronal-Immune Activities

Evidence has pointed to immune dysregulation in TS; however, the mechanistic details of this pathophysiology remain obscure. Studies on antineuronal antibodies, cerebrospinal fluid (CSF), or brain tissue may provide more information on the investigation of how immune dysregulation contributes to the pathogenesis of TS and the extent of neuroinflammation.

#### 3.3.1. Alteration in Microglia

In a transcriptome analysis of the basal ganglia in postmortem brains from nine patients with TS, Lennington et al. [78] described the upregulation of immune-related genes as well as increased CD45^+^ microglial expression, indicating microglial activation in the striatum. However, an important caveat is that this study was performed in adult refractory TS, which is a severe form of TS. In line with this report, by using positron emission tomography imaging with (11)C-[R]-PK11195 (PK), a ligand that binds to the transporter protein expressed by activated microglia, Kumar et al. [79] presented increased PK binding in the caudate nuclei bilaterally in children with PANDAS and TS. This indicated that localized inflammatory microglial activation occurred in the striatum in patients with TS. Microglia, the resident immune cells of the central nervous system, were also found to have an important role in neuronal survival as well as the maintenance of neurogenesis [80,81]. Animal studies may provide insight into the microglial-related pathomechanism in TS. The *Hdc*-knockout (KO) mouse model, which recapitulates the human TS phenotype, has shown normal expression of inflammatory markers but reduced arborization of microglia as well as a reduced number of microglia expressing insulin-like growth factor 1 (IGF1) in the basal condition [82], suggesting an impairment of protection to neurons, as IGF1-expressing microglia are essential for neuronal survival and neurogenesis [80]. However, an inflammatory challenge with bacterial lipopolysaccharide (LPS) dramatically resulted in microglial activation in the striatum and enhanced induction of proinflammatory cytokines (TNF-*α* and IL-1*β*) in *Hdc*-KO mice compared to wild-type controls. These findings in the mouse model suggest that there may be deficits in microglia-mediated neuroprotection, along with overreactivity to environmental challenges in TS [82,83].

#### 3.3.2. Antineuronal Antibodies

SC and PANDAS may share an underlying pathogenesis. Accumulating evidence shows that autoantibodies target antigens in the basal ganglia of these patients [84,85,86,87,88]. Through the molecular mimicry process, autoantibodies against N-acetyl-beta-D-glucosamine (GlcNAc), the dominant epitope of the GAS carbohydrate, cross-react with neuronal antigens, leading to neurological symptoms [85,89]. Cross-reacting neuronal antigens include lysoganglioside-GM1 and beta-tubulin, both of which immunologically mimic GLcNAc [84,85,86]. They further induced CaMKII kinase activation [84,85]. CaMKII, an enzyme highly expressed in the brain, plays a pivotal role in the regulation of glutamatergic synapses and is important in neuronal signaling cascades and synthesis of neurotransmitters, including dopamine. It has an important function in behavior, learning, and memory [90]. Therefore, CaMKII dysfunction may be partly related to behavioral issues such as anxiety, OCD, and emotional liability in these patients.

Autoantibodies targeting dopamine receptors may also play an important role in the pathogenesis of SC and PANDAS. However, the findings have been inconsistent, which might be due to the different antibody-detection techniques (i.e., enzyme-linked immunosorbent, Western blot, and cell-based assays) applied in the different studies. Using cell-based assays, Dale et al. [87] detected surface dopamine-2 receptor (D2R) IgG antibodies in the sera of 30% of patients with SC in comparison with 9% of patients with TS but none in patients with PANDAS and the controls. No dopamine-1 receptor (D1R) IgG was detected in the disease or control groups. However, subsequent studies have shown that anti-D1R and anti-D2R were significantly elevated in patients with active SC and PANDAS [86,88]. The anti-D2R/anti-D1R ratio is correlated with the severity of neuropsychiatric symptoms in SC [88].

The findings from animal models provide further support for the streptococcal autoimmune hypothesis, as a passive transfer of antistreptococcal antibodies into murine models led to behavioral alteration and characteristic behaviors of both PANDAS and SC [91]. Antistreptococcal antibodies were deposited in the striatum with specific brain proteins such as dopamine receptors and serotonin transporters in in-vitro studies; the antibodies also reacted with D1 and D2 dopamine receptors and 5HT-2A and 5HT-2C serotonin receptors [91].

However, evidence that supports antineuronal antibodies in TS has been vague [92,93] (Table 1). Wenzel et al. [25] observed positive CSF OCB in 20–38% of adults with TS, indicating intrathecal antibody synthesis, but the same group failed to detect any specific antineuronal antibody in the CSF in their subsequent study [25,94]. In contrast to the findings in adult TS, an exploration study measured arrays of immune cells, cytokines, OCB, and other IGs on the CSF of children with TS, and they did not show significant difference regarding immune cell subsets, cytokines, or chemokines. Additionally, none of the patients had positive CSF OCB [95]. In a large-scale study involving children with TS of different stages, only two children had antibodies binding to the N-methyl-D-aspartate receptor. No other specific antibodies were detected. Therefore, current evidence supports the role of pathogenic antibodies in the pathogenesis of TS. However, as only specific antigens of interest have been screened, further screening for other specific antigens should be performed.

## 4. Animal Models for Immune Dysregulation in TS

TS is a multifactorial neurological disorder with a broad spectrum of clinical presentations. With the increase of evidence of immunological involvement in TS pathogenesis, animal models are another effective tool to clarify the role of each cytokine or autoantibody in TS in a relatively well-controlled environment. The main strategies to developing an immune-mediated animal model of TS can be divided into four categories: (1) injection of cytokines or other immune mediators that are already known to have pathogenic roles in TS; (2) immunization with specific microorganisms to induce autoantibodies that may target TS in relevant parts such as the CSTC circuit; (3) passive transfusion with sera derived from affected patients or immunized animals that contain autoantibodies to interrupt central nervous system signal transmission; (4) genetically modified mice that may produce autoantibodies or be more susceptible to induced tic disorders under certain environmental stimuli (Table 2) [96].

### 4.1. Injection of Cytokines or Other Immune Mediators

Previous studies in patients with TS have observed an increased level of inflammatory cytokines, including TNF-α and ILs, such as IL-1 beta, IL-2, IL-6, IL-8, IL-12, IL-17, and INF-γ-induced protein 10 [24,115,116]. Mice treated with IL-2 showed increased norepinephrine utilization in the hypothalamus and enhanced dopamine activity in the prefrontal cortex. IL-6 increased the activity of serotonin (5-HT) and dopamine in the hippocampus and prefrontal cortex, whereas IL-1 might induce multiple central monoamine alterations and increase plasma corticosterone levels [97]. Mice treated with IL-2 and IL-6 showed more frequent digging, rearing, and grooming and spent more time in ambulatory or nonambulatory exploration [98]. Moreover, IL-2 induced excessive climbing behavior that could be blocked by the dopamine D1 receptor antagonist and the dopamine D2 receptor antagonist [117]. Perinatal IL exposure may also influence the behavior of offspring. The offspring of IL-2-induced pregnant autoimmune disease-sensitive mice had increased self-grooming and eye-blinking [99]. IL-6 injection of mid-gestational pregnant mice resulted in deficits of prepulse inhibition in the offspring. Patients with TS also usually have abnormal performance in this sensorimotor process [100]. Soluble cytokine receptors are natural components in body fluids. Injection of soluble IL-2 receptors alpha and beta induced an increase in ambulatory behaviors and stereotypic movement with increased neuronal activities in the cortex and striatum [101]. On the contrary, the soluble IL-6 receptor could localize in brain regions related to the CSTC circuit and induce repetitive stereotypies, which had high similarity to patients with TS [102]. The above results suggested that excessive cytokine levels may result in clinical symptoms similar to TS. Therefore, TS may be related to some kinds of chronic inflammatory process.

### 4.2. Immunization with Specific Microorganism

The correlation between TS and GAS-infection-related autoimmunity is well known. Hoffman et al. [103] established an animal model for neuropsychiatric diseases associated with GAS infection. Increased immunity response was observed in the deep cerebellar nucleus, thalamus, and global pallidus. These autoantibodies also had cross-reactions with complement C4 protein and alpha2-macroglobulin in the brain. Behavioral test results of these mice revealed increased rearing, submission, and defensive-escape behaviors and decreased environmental exploration [104]. Passive infusion of sera from GAS-infected mice to naïve mice can also induce similar behavioral abnormality, with increased IgG deposit in the hippocampus and periventricular area [104]. Ajmone-Cat et al. [105] applied environmental psychosocial stress to a GAS-infected mouse model and revealed altered cytokine expression, including IL-1β, TNF-α, and IL-10, and immune-related enzyme expression in the hippocampus and hypothalamus, with impaired mitochondrial function. This result also suggested the correlation between environmental factors and the autoimmune response.

### 4.3. Passive Sera Transfusion

Passive administration of antineuronal antibodies derived from patients with TS or related disorders into rodent striatum is another method to test the immunologic hypothesis in TS. Hallett et al. [106] performed intrastriatal microinjections of sera derived from patients with TS into rats. Increased motor stereotypies and episodic vocalization were recorded. In addition, with a similar approach, Taylor et al. [107] found that the severity of oral stereotypies is highly increased in rats injected with high autoantibody titers. Increased tic-like behaviors, including motor stereotypies, genital grooming, and forepaw grooming, were also mentioned in other studies employing intrastriatal microinjections of sera from patients with TS [118,119]. However, behavioral alteration after intrastriatal microinjection with sera or autoantibodies derived from patients with TS was not consistently reported. Loiselle et al. [108] chose sera from patients with TS or PANDAS, and Ben-Pazi et al. [109] used antibasal antibodies from patients with SC. Both studies did not find a difference in behavioral change or immunohistology staining for dopaminergic or GABAergic markers. The reasons for failure to distinguish these two groups might be related to the injection of nonpathogenic autoantibodies or inadequate autoantibody titers. These inconsistent results may reflect the complexity of immunological interaction in patients with TS. The influence of autoantibodies against striatum could not explain the whole pathogenesis of immunological response in TS; thus, some other systemic factors should be further evaluated. Besides intrastriatal microinjections, Zhang et al. injected anti-GAS monoclonal IgM and IgG subcutaneously in a mouse model [110]. Antistreptococcal IgG stimulated vertical activities and ambulation, and antistreptococcal IgM increased stereotypies like head bobbing, sniffing, and grooming. Antistreptococcal IgM also induced Fos-like immunoactivities in CSTC structures, including the caudate nucleus, nucleus accumbens, and the motor cortex. This result also suggests the different roles of specific IgM and IgG in TS pathogenesis.

### 4.4. Transgenic Animal Models

Some genetically modified mouse strains may present altered immunological function or have a higher risk of autoimmune diseases. Histidine decarboxylase (*Hdc*)-knockout mice were developed nearly 20 years ago [120]. *Hdc*-knockout mice cannot synthesize histamine, which is a biogenic amine with an important role in inflammation regulation. *Hdc*-knockout mice have increased stereotypies and elevated repetitive behaviors, such as grooming after stress or psychostimulant challenge [111,112]. This phenomenon could also be observed in patients with TS. Besides the dysregulation of dopamine levels [53,111], *Hdc*-knockout mice have a normal number of microglia but reduced ramifications [82]. Some studies have suggested that histamine-stimulated microglia may have neuroprotective effects from the inflammatory process induced by some stimuli such as lipopolysaccharides [121]. The above results suggest that histamine is one of the key factors in TS pathogenesis through the dysregulation of the dopamine level and microglia in the neuroinflammatory process [53]. The *Hoxb8* gene is another candidate for transgenic mice in TS studies. *Hoxb8* is involved in the differentiation of bone-marrow-derived microglia. *Hoxb8*-knockout mice developed excessive grooming behavior and hair loss [113,114]. This abnormal behavior could be treated after bone marrow transplantation from wild-type mice, and the excessive grooming behavior could be induced after transplantation of bone marrow from *Hoxb8*-knockout mice into wild-type mice [114]. Since *Hoxb8* is only present in approximately 40% of microglia, it is still unclear whether *Hoxb8*-related microglia have an important function in the maintenance of normal behavior or *Hoxb8*-negative microglia result in abnormal grooming in *Hoxb8*-related behavioral disorders.

## 5. Conclusions

**Figure 1:**
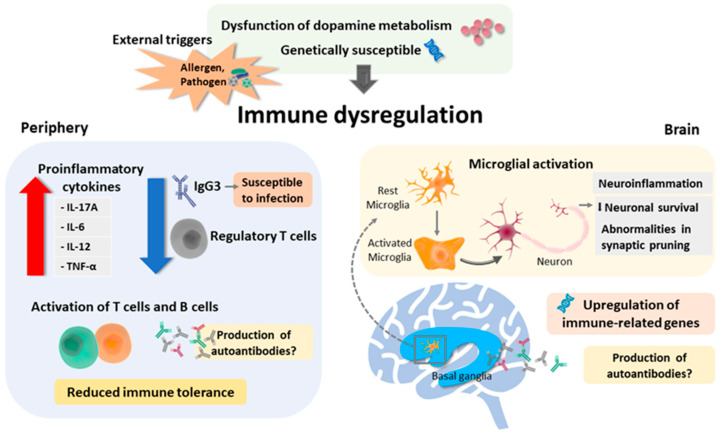
Summary of the possible underlying mechanism leading to immune dysfunction in Tourette syndrome. Predisposing conditions include dysfunction of dopamine metabolism (hyperdopaminergic state) and genetic susceptibility. External triggers such as allergen and pathogen may also, in part, facilitate periphery and brain immune dysregulation. In the periphery, reduced regulatory cells (which might be facilitated by the hyperdopaminergic state), increased release of proinflammatory cytokines, and activation of T-cells and B-cells may lead to reduced immune tolerance. Additionally, the decreased IgG3 level may lead to a defective immune response to pathogens, hence resulting in persistent inflammation. In the brain, regulation of immune-related genes in localized inflammatory microglial activation in the striatum in TS may result in neuroinflammation and additionally affect neuronal survival and abnormalities in neurogenesis. However, the production of any specific pathogenic autoantibody remains to be explored.

Currently, TS is considered a multifactorial neurological developmental disorder, and the aforementioned studies have suggested the role of immunological dysfunction in the pathogenesis of TS. The clinical correlation with GAS infection, autoantibody analysis, and gene expression studies all support immunological involvement in TS. The dysregulation of neurotransmitters such as serotonin, acetylcholine, GABA, or dopamine not only affects the function of the CSTC circuit directly but also influences immune systems indirectly. Animal studies have provided a relatively well-controlled environment for an autoimmunological study in TS and revealed evidence of immunological involvement. The summary of the possible underlying mechanisms leading to immune dysfunction in TS based on current evidence is presented in Figure 1. Although the basis of the pathogenesis of TS is still not understood, immunological dysregulation may provide a different viewpoint to find the ultimate cause of TS.

## Figures and Tables

**Table 1:** Studies of antineuronal antibody and CSF immunoglobulin in tic disorders and Tourette syndrome.

Reference	Method	Specimen	Main Findings
[87]	CBA	Serum	Elevated D2R IgG in 4/44 (9%)
[92]	CBA	Serum	In a pediatric TS cohort of 30 siblings at preclinical and onset, 158 at chronic phase, 2 had NMDAR Ab weak positive, no other specific Abs (NMDAR, CASPR2, LGI1, AMPAR, and GABAAR) were detected; found Ab reactive with brain tissue, mainly to the hippocampus, the basal ganglia or the cerebellum in 12%
[93]	CBA	Serum	In 51 adult TS, no specific Abs (CASPR2, LGI1, NMDAR, AMPA1, AMPA/2, or GABAB1/B2) were detected
[25]	IF	CSF	Presence of OCB in 38% (8 of 21) adult TS
[94]	IF, CBA	CSF	Presence of OCB in 20% (4 of 20) adult TS; did not detect Abs to any of the antigens on SY5Y neuronal stem-cells and astrocytes cultures
[95]	IF	CSF	In 5 pediatric TS, none were detected for OCB and other immunoglobulins

Abbreviation: AMPAR: α-amino-3-hydroxy-5-methyl-4-isoxazolepropionic acid receptor; Abs: antibodies; CASPR2: contactin-associated protein 2; CBA: cell-based assay; CSF: cerebrospinal fluid; D2R: dopamine-2 receptor; GABAAR: Gamma-aminobutyric acid type A receptor; GABAB1/B2: Gamma-aminobutyric acid B1/B2 receptor; IF: isoelectric focusing; IgG: immunoglobulin G; LGI1: leucin-rich glioma-inactivated protein; NMDAR: N-methyl-D-aspartic acid receptor; OCB: oligoclonal band; TS: Tourette syndrome.

**Table 2:** Findings of immunological involvement in animal models.

**Cytokine Injection**			
**Reference**	**Method**	**Animal Type**	**Main Finding**
[97]	Treated with IL-1, IL-2, and IL-6	Mouse	IL-1: multiple central monoamine alteration and increased plasma corticosterone level IL-2: increased NE utilization and enhanced DA activity IL-6: increased activity of 5-HT and DA
[98]	Treated with IL-2 and IL-6	Mouse	Increased digging, rearing, grooming and more ambulatory or nonambulatory exploration
[99]	Prenatal exposure with IL-2	Mouse	Increased self-grooming and eye-blinking
[100]	Prenatal exposure with IL-6	Mouse	Deficits of prepulse inhibition test
[101]	Injection of soluble IL-2 receptors	Mouse	Increased ambulatory behaviors and stereotypies Increased neuronal activities over cortex and striatum
[102]	Injection of soluble IL-6 receptors	Mouse	Induced repetitive stereotypies
**Immunization with Specific Microorganism**			
**Reference**	**Method**		**Main Finding**
[103]	Immunized with GAS homogenate	Mouse	Increased immunity response was observed in deep cerebellar nucleus, thalamus, and global pallidus
[104]	Immunized with GAS homogenate	Mouse	Increased rearing, submission, and defensive-escape behavior and decrease environmental exploration
[105]	Environmental psychosocial stress for GAS-infected mouse	Mouse	Altered cytokine, including IL-1β, TNF-α, IL-10 Impaired mitochondrial function
**Passive Sera Transfusion**			
**Reference**	**Method**		**Main Finding**
[106]	Intrastriatal microinjection	Rat	Increased motor stereotypies and episodic vocalization
[107]	Intrastriatal microinjection	Rat	Increased tic-like behaviors
[108,109]	Intrastriatal microinjection	Rat	No difference in behavioral change or immunohistology staining between experimental and control groups
[110]	Intravenous injection	Mouse	Antistreptococcus IgG stimulated vertical activities and ambulation Antistreptococcus IgM induced Fos-like immunoactivities in CSTC structures
**Transgenic Animal Model**			
**Reference**	**Method**		**Main Finding**
[52,53,111,112]	*Hdc*-knockout mice	Mouse	Increased stereotypes elevated repetitive behaviors Dysregulated dopamine level and microglia in neuroinflammatory process
[113,114]	*Hoxb8*-knockout mice	Mouse	Excessive grooming behaviors and hair loss Abnormal behaviors could be treated with bone marrow transplantation from wild-type mice

Abbreviation: 5-HT: serotonin; CSTC: corticostriato–thalamocortical; DA: dopamine; GAS: group A streptococcus; Hdc: histidine decarboxylase; IgG: immunoglobulin G; IgM: immunoglobulin M; IL: interleukin; NE: norepinephrine; TNF-α: tumor necrosis factor-α.

## Data Availability

Data sharing not applicable.
